# Dynamic Neural Network Changes Revealed by Voxel-Based Functional Connectivity Strength in Left Basal Ganglia Ischemic Stroke

**DOI:** 10.3389/fnins.2020.526645

**Published:** 2020-09-18

**Authors:** Qiong-Ge Li, Cheng Zhao, Yi Shan, Ya-Yan Yin, Dong-Dong Rong, Miao Zhang, Qing-Feng Ma, Jie Lu

**Affiliations:** ^1^Department of Radiology, Xuanwu Hospital, Capital Medical University, Beijing, China; ^2^Beijing Key Laboratory of Magnetic Resonance Imaging and Brain Informatics, Beijing, China; ^3^Department of Neurology, Xuanwu Hospital, Capital Medical University, Beijing, China; ^4^Department of Nuclear Medicine, Xuanwu Hospital, Capital Medical University, Beijing, China

**Keywords:** ischemic stroke, connectivity strength, basal ganglia, resting state, fMRI

## Abstract

**Objective:**

This study intends to track whole-brain functional connectivity strength (FCS) changes and the lateralization index (LI) in left basal ganglia (BG) ischemic stroke patients.

**Methods:**

Twenty-five patients (*N* = 25; aged 52.73 ± 10.51 years) with five visits at <7, 14, 30, 90, and 180 days and 26 healthy controls (HCs; *N* = 26; 51.84 ± 8.06 years) were examined with resting-state functional magnetic resonance imaging (rs-fMRI) and motor function testing. FCS and LI were calculated through constructing the voxel-based brain functional network. One-way analysis of covariance (ANOVA) was first performed to obtain longitudinal FCS and LI changes in patients among the five visits (Bonferroni corrected, *P* < 0.05). Then, pairwise comparisons of FCS and LI were obtained during the five visits, and the two-sample *t* test was used to examine between-group differences in FCS [family-wise error (FWE) corrected, *P* < 0.05] and LI. Correlations between connectivity metrics (FCS and LI) and motor function were further assessed.

**Results:**

Compared to HCs, decreased FCS in the patients localized in the calcarine and inferior occipital gyrus (IOG), while increased FCS gathered in the middle prefrontal cortex (MPFC), middle frontal gyrus, and insula (*P* < 0.05). The LI and FCS of patients first decreased and then increased, which showed significant differences compared with HCs (*P* < 0.05) and demonstrated a transition at the 30-day visit. Additionally, LI at the third visit was significantly different from those at the other visits (*P* < 0.05). No significant longitudinal correlations were observed between motor function and FCS or LI (*P* > 0.05).

**Conclusion:**

Focal ischemic stroke in the left BG leads to extensive alterations in the FCS. Strong plasticity in the functional networks could be reorganized in different temporal dynamics to facilitate motor recovery after BG stroke, contribute to diagnosing the disease course, and estimate the intervention treatment.

## Introduction

Cerebral stroke is a major cause of mortality and long-term disability. More than 80% of strokes stem from ischemic damage to the brain due to the acute reduction of the blood supply (Kessner et al., 2019). The region of basal ganglia (BG), which is a predilection site of stroke, is a neuroimaging term, including anatomical structures of the amygdala, caudate nucleus, lentiform nucleus, claustrum, capsula interna, and the surrounding white matter (Kreitzer and Malenka, 2008; Florio et al., 2018). The BG control voluntary motor activity primarily by regulating the motor and premotor cortex, and BG ischemic stroke could potentially have a long-term effect on movement (Qureshi et al., 2003**;** Guo et al., 2017). Therefore, the exploration of the mechanism of BG stroke can contribute to providing potentially useful metrics for monitoring the efficacy of rehabilitation therapy in these patients.

Resting-state functional magnetic resonance imaging (rs-fMRI) has exhibited unique advancements in non-invasively exploring spontaneous functional connectivity (FC) alterations in brain disorders (Biswal et al., 2010). Previous FC methods are useful in evaluating connectivity patterns for specific brain regions or distinct components of the brain network but do not reflect the whole connectivity pattern in all brain elements. In contrast, functional connectivity strength (FCS), a graph theory-based analysis method can explore nodal weighted centrality and reflect its FC within the whole brain network (Gao et al., 2017**;** Zhang et al., 2020). This measurement indirectly reflects the position and importance of the node or brain regions in the whole-brain networks, without ***a priori*** defined seed (Wang L. et al., 2014). Thus, it is highly suitable for studying diseases with unclear pathological mechanisms and works as an effective index. Moreover, lateralization is a critical factor for estimating the changes in motor function and could be assessed based on rs-fMRI. In previous stroke investigations, the measurement of lateralization was used to reflect the degree to which motor change processes are lateralized (Marangon et al., 2014). Combination of rs-fMRI and lateralization may provide reliable metrics and has been successfully used in brain disorders (Rosazza et al., 2013).

Although abundant rs-fMRI studies of stroke have been reported on large-scale brain lesions and provided valuable content, lesion location is still an important factor in exploring brain function. More stroke studies with lesions in isolated brain regions are needed to address these issues (Park et al., 2016; Rondina et al., 2016; Desowska and Turner, 2019). As mentioned above, BG stroke could cause long-term motor impairment, but the mechanism of its brain function has rarely been studied and not been elucidated. A previous longitudinal study employing task-based fMRI has demonstrated that the contralateral sensorimotor cortex (SMC) appears to be involved in functional rehabilitation following BG ischemic stroke (Fu et al., 2016). However, comprehensive and integrated analysis of BG stroke has not been conducted.

**FIGURE 1:**
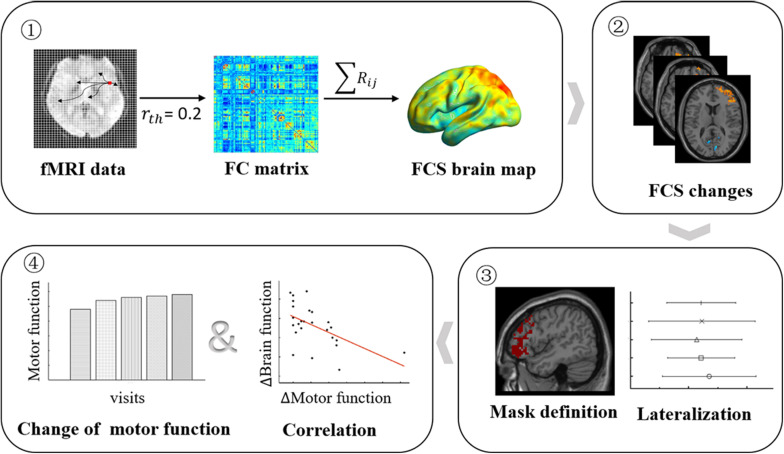
Longitudinal study design flowchart. Patients underwent five (<7, 14, 30, 90, and 180 days after stroke onset) MRI scans and clinical motor function assessments, while HCs underwent a functional MRI (fMRI) scan. **Step 1**: the FCS brain map was derived for each participant and at each visit. **Step 2**: the FCS changes between patients at each visit, and HCs were obtained by the two-sample *t* test model. **Step 3**: the significant brain regions of FCS changes were submitted, and mirror superposition was used as a mask to calculate LI. **Step 4**: the changes in FCS and LI and changes in motor function were used to perform brain–behavior association evaluations. HCs, healthy controls; FCS, functional connectivity strength; LI, lateralization index.

Here, our study aims to carry out a long-term follow-up of rs-fMRI in the left BG stroke patients to characterize the neural mechanism of motor recovery after stroke. First, we performed whole-brain FCS analysis to assess brain regions exhibiting intrinsic functional network changes compared to healthy controls (HCs) at the voxel level. Second, we investigated temporally evolving functional lateralization based on brain regions of FCS changes. Finally, we explored the associations of altered functional lateralization with clinical outcomes in stroke patients. The design flowchart of this study is shown in Figure 1. In this study, we expected FCS changes in patients compared to HCs to improve with motor recovery, and lateralization changes may show trajectory in sync with FCS.

## Materials and Methods

### Participants

Twenty-five patients (39–73 years old; mean age, 52.73 ± 10.51 years; male, 18) with isolated left BG ischemic stroke were recruited. The criteria for inclusion included (a) first-onset stroke and diagnosis by neurological testing, (b) five MRI scans and motor function (<7, 14, 30, 90, and 180 days after stroke onset), (c) single lesion location confined to the left BG (region of the BG was confined to the BG nucleus and its surrounding white matter), (d) complete admission history, and (e) no previous stroke and no other intracranial lesion or mental disease. The exclusion criteria included (a) contraindication for MRI, (b) unclear onset of symptoms, (c) recurrent and secondary stroke during visit, and (d) blindness or/and deafness, aphasia, or visual field deficit. Twenty-six age- and sex-matched HCs (30–70 years old; mean age, 51.84 ± 8.06 years; male, 15) were recruited and underwent MRI scanning, and MRI data from HCs were acquired along with the baseline of patient group. Each participant was right-handed. It should be mentioned that we included only participants with Fazekas scores under 2, whose white matter abnormalities could be considered as normal aging changes (Fazekas et al., 1987). This study was approved by the local Institutional Review Board, and written informed consent was obtained from all participants. The data that support the findings of this study are available from the corresponding author upon reasonable request.

### MRI Data Acquisition

Magnetic resonance imaging scans were conducted on a Siemens 3.0 Tesla Tim Trio scanner using a 12-channel head coil. Resting-state images were acquired using echo planar imaging (EPI) sequence with repetition time (TR)/time to echo (TE) = 3,000/30 ms, flip angle = 90°, and isotropic voxel size = 3 mm × 3 mm × 3 mm. Resting-sate fMRI of each participant involved two or four runs, and each run lasted 360 s and required 124 volumes. To maintain the steadiness and uniformity of resting-state fMRI data, this study selected the second fMRI run for subsequent research. In addition, we assessed high-resolution three-dimensional (1 mm^3^) T1-weighted magnetization-prepared rapid gradient-echo (MP-RAGE) images and T2-weighted images (slice thickness = 5 mm).

### Motor Function

All eligible patients received comprehensive motor examination, which included Fugl-Meyer Assessments (FMA) and right-hand grip strength. The FMA scale, as a remarkable motor domain, was tested. FMA scores were evaluated using 33 tasks that can quantitatively reflect motor function, balance, sensation, and joint function of the upper limbs. Thirty-three scores were summed and then normalized to a score between 0 and 100 to represent the FMA score (Fugl-Meyer et al., 1975; Guo et al., 2016). Grip strength was investigated with a mechanical dynamometer. Patients performed two trials with the right hand, and the mean value was calculated for right hand individually.

### Lesion Probability Map

**FIGURE 2:**
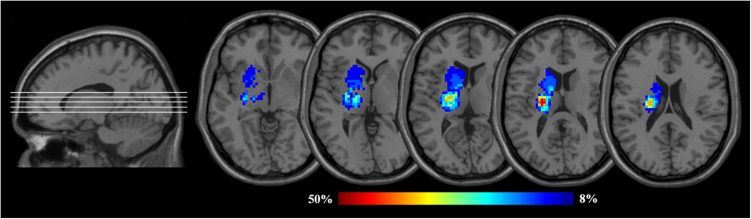
Lesion probability maps for the left BG patients. The heat maps corresponding to the probability of patients having a lesion in that area overlaid on axial slices from a standard template in MNI space (MNI152). BG, basal ganglia; MNI, Montreal Neurological Institute. Images are presented in neurological convention (i.e., the left hemisphere is on the left side).

As shown in Figure 2, lesions of all patients at baseline were outlined on T2-weighted images using tools from MRIcron software^1^, and the lesion probability map was then created using Statistical Parametric Mapping (SPM12). The corresponding procedures were as follows: (a) lesions were resliced to individual T1-weighted images; (b) the resliced lesions were smoothed with 3-mm full-width half-maximum (FWHM) Gaussian kernel; (c) linearly registered and normalized to Montreal Neurological Institute (MNI) space were performed; (d) the resulting transformation lesions were coregistered and averaged, resulting in maps with a value for each voxel ranging from 0 to 1, indicating the proportion of patients with a lesion in that particular voxel.

### Image Preprocessing

All rs-fMRI data were preprocessed using SPM12 and Data Processing Assistant for rs-fMRI (DPARSF) (Yan et al., 2016). Preprocessing of rs-fMRI data included removing the first four volumes to make the magnetization approach a dynamic equilibrium, slicing timing to the median reference slice, and realigning for head motion correction. Then, the data were spatially normalized to the MNI space using an echo-planar imaging (EPI) template with a voxel size of 3 mm × 3 mm × 3 mm. Several nuisance variables, including Friston’s 24 head-motion parameters (Friston et al., 1994) and averaged signals from white matter and cerebrospinal fluid tissue, were removed using multivariate linear regression analysis. Finally, 0.01–0.1 Hz bandpass temporal filtering was used to remove low-frequency magnetic field drifts and high-frequency respiratory and cardiac noise.

### Voxel-Based Functional Connectivity Strength

For the calculation of voxel-based FCS, we first constructed a voxel-based FC matrix by calculating Pearson’s correlation coefficients (r) between all pairs of voxels within a gray matter mask (number of voxels = 45,381). Here, we took each voxel as a node and estimated Pearson’s correlation (*r* value) between any pair of voxels as the internodal edge weight (Buckner et al., 2009; de Pasquale et al., 2018). The individual correlation matrices were further translated to a z-score matrix using the Fisher r-to-z approach. To eliminate low temporal correlations resulting from signal noise (Wang C. et al., 2014; Liu W. et al., 2015), we conservatively restricted our explorations to positive correlations and defined an internodal correlation threshold of *r* = 0.2. Finally, the measurement of FCS for each given voxel was computed in the z-score matrix, by the following equation

$$D_{i}=\sum_{j=1}^{N}r_{\mathrm{ij}}\left(i\neq j\right)$$

where *D*_*i*_ is FCS for a given voxel i, *r*_ij_ is a connection or edge weight between voxel *i* and voxel *j*, and *N* is the number of voxels in the GM mask. FCS maps of all participants were obtained.

To improve normality, the raw FCS value was then standardized to z scores using the following equation

$$z_{i}=\frac{D_{i}-\bar{D}}{\delta_{D}}\left(i=1\ldots \ldots N\right)$$

where $\bar{D}$ and δ_*D*_ represents the mean FCS and standard deviation across whole-brain voxels, respectively.

### Lateralization Index Calculation

Brain regions with significant FCS changes between the patients with five visits (<7, 14, 30, 90, 180 days) and HCs were first extracted and summed and then binarized as a mask. Mirror superposition was finally used in the mask, which was symmetrically categorized into the left and right hemispheres. Then, the LI of each participant was calculated using the following equation

$$\mathrm{LI}=\frac{\left({\mathrm{FCS}}_{\mathrm{left}}-{\mathrm{FCS}}_{\mathrm{right}}\right)}{{\mathrm{FCS}}_{\mathrm{left}}+{\mathrm{FCS}}_{\mathrm{right}}}$$

where FCS_left_ and FCS_right_ represent the sum of FCS values of all left and right hemisphere voxels contained in the mask. LI ranged from −1.0 (means right FCS only) to 1.0 (means left FCS only), and LI values close to 0 indicated symmetrical FCS.

### Statistical Analysis

The age of patients and HCs was compared by a two-sample *t* test. The sex composition of the two groups was compared using a chi-square test (two-tailed). Additionally, a one-way analysis of covariance (ANOVA) was used to assess differences in FMA and grip strength among five visits of the patient group (Bonferroni corrected, *P* < 0.05). Then, a paired *t*-test was applied to measure detailed FMA and grip strength changes between patients at the last four visits and baseline.

To explore differences in FCS, a one-way repeated measure ANOVA analysis and *post hoc* test were first performed to obtain longitudinal changes in patients among five visits (Bonferroni corrected, *P* < 0.05). Then, the two-sample *t* test was used to examine FCS differences between the patients and HCs. After 5,000 non-parametric random permutations, threshold-free cluster enhancement (TFCE) (Smith and Nichols, 2009) was used to make family-wise error correction (FWE, *P* < 0.05) for each FCS contrast. The statistical analysis of FCS was performed using SPM12.

Like the statistical methods of FCS, one-way repeated measure ANOVA analysis and *post hoc* test were also used to obtain longitudinal LI changes in patients among five visits (Bonferroni corrected, *P* < 0.05). LI differences between the patients and HCs were also tested with two-sample *t* test. SPSS 21.0 was used to perform the above statistical tests.

Finally, relationships between changes of connectivity metrics (ΔFCS and ΔLI) and corresponding changes in motor variables (ΔFMA and Δgrip strength) in patients were further assessed by using the partial correlation method. Δ refers to differences of those measures (FCS, LI, FMA, and grip strength) between the baseline and the remaining each visit. We controlled age and sex in the analyses.

### Validation Analysis

To estimate whether these results are robust, we performed this validation test. Given the influence of different thresholds on brain network topology construction (Buckner et al., 2009), we selected another threshold (*r* = 0.4) to calculate the corresponding FCS and LI to verify the results. Intragroup and between-group FCS and LI changes were re-examined using ANOVA and two-sample *t* test (see Supplementary Material).

## Results

### Demographics and Clinical Characteristics

**FIGURE 3:**
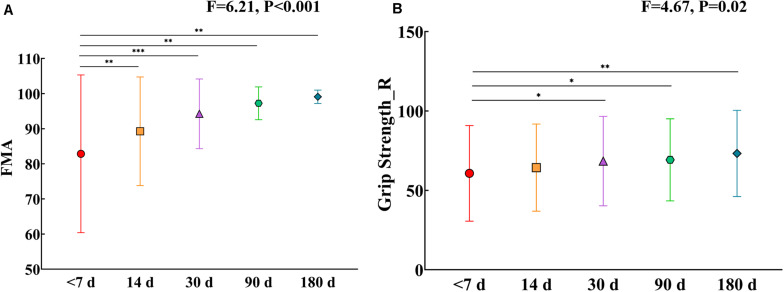
**(A)** FMA changes in patients after five visits. **(B)** Grip strength changes in patients after five visits. Error bars show standard deviations. *, *P* < 0.05; **, *P* < 0.01; ***, *P* < 0.001. FMA, Fugl-Myer Assessment; R, Right hand.

Table 1 lists the demographic and clinical information for both stroke patients and HCs. Twenty-five patients with left BG stroke and 26 HCs were well matched in age and sex. Moreover, significant FMA (*F* = 6.21, *P* < 0.001) and grip strength (*F* = 4.67, *P* = 0.02) differences in patients between the first visit and the other four were observed. As shown in Figure 3, it should be noted that FMA and grip strength showed significant improvement.

**TABLE 1:** Demographic characteristic for patients with left BG infarction and HCs.

**Parameter**	**Patients (*n* = 25) Mean ± SD**	**HCs (*n* = 26) Mean ± SD**	***t*/χ^2^**	***P***
Age (years old)	52.73 ± 10.51	51.84 ± 8.06	0.34	0.73^a^
Sex (male/female)	18/7	15/11	1.06	0.30^c^
FMA				
<7 days	82.85 ± 22.46			
14 days	89.27 ± 15.47		3.55	0.00^b^
30 days	94.25 ± 9.93		4.07	0.00^b^
90 days	97.25 ± 4.67		3.54	0.00^b^
180 days	99.09 ± 1.91		3.79	0.00^b^
Grip strength_R				
<7 days	60.69 ± 6.02			
14 days	64.31 ± 5.48		1.76	0.09^b^
30 days	68.40 ± 5.63		2.29	0.03^b^
90 days	69.23 ± 5.17		2.17	0.04^b^
180 days	73.23 ± 5.42		2.83	0.01^b^

*BG, basal ganglia; HCs, healthy controls; SD, standard deviation; FMA, Fugl-Myer Assessment; R, right hand. ^*a*^The *P* value was obtained by the two-sample *t* test. ^*b*^The *P* value was obtained by the paired *t* test. ^*c*^The *P* value was obtained by the chi-square test.*

### FCS Changes Between the Patients and HCs

**FIGURE 4:**
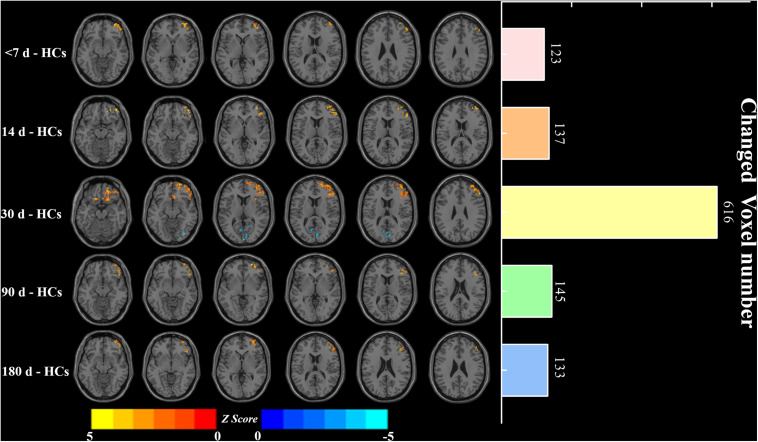
Significant FCS differences and changed voxel numbers between patients at five visits of <7, 14, 30, 90, and 180 days and HCs. Axial maps of each row represent the results of each contrast. Values are expressed as z scores, where hot colors represent increased FCS and cold colors represent decreased FCS in the left BG stroke patients. Each item in the right bar graph corresponds to each row in the left axis map. FCS, functional connectivity strength; HCs, healthy controls.

**FIGURE 5:**
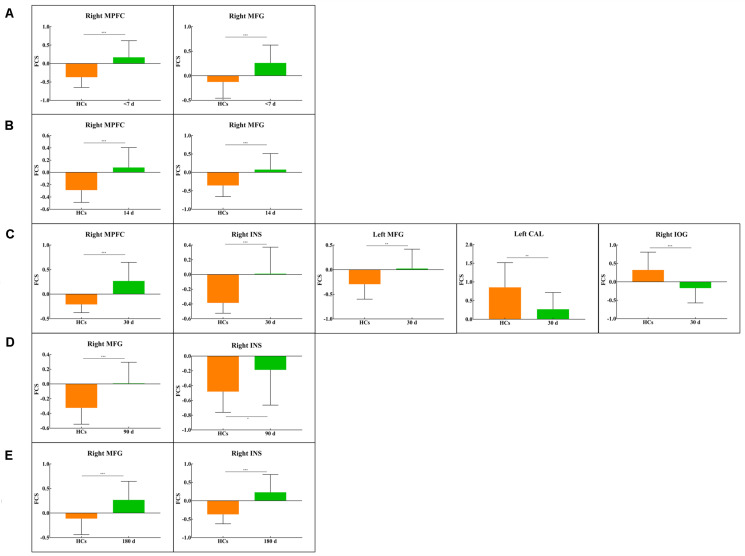
Bar plots show FCS values for all clusters in each between-group comparison: **(A)** <7 d – HCs. **(B)** 14 d – HCs. **(C)** 30 d – HCs. **(D)** 90 d – HCs. **(E)** 180 d – HCs. The bar height indicates the mean value, and the error bar indicates the standard deviation for a given group. MFG, middle frontal gyrus; MPFC, medial prefrontal cortex; CAL, calcarine; INS, insula; IOG, inferior occipital gyrus. *P < 0.05; **P < 0.01; ***P < 0.001.

First, the results showed that there was no significant FCS difference among the different visits in the patient group using repeated measure one-way ANOVA (*P* > 0.05). We next examined the between-group FCS changes, and Figure 4 shows clusters with significantly altered FCS between the patients at each visit and the HCs at baseline. Compared to HCs, the patients at baseline exhibited significantly increased FCS in the right middle prefrontal cortex (MPFC) and right middle frontal cortex (MFG). Similarly, patients at the <14 days visit also showed significantly increased FCS in the right MPFC and right MFG, while changes in voxel numbers in the second visit (*N* = 123) were less than those of the baseline (*N* = 137). Furthermore, the patients at the <30 days visit showed significantly increased FCS in the right MPFC, right insula (INS), and left MFG, while significant decreases were observed in the left lingual gyrus (LING) and right inferior occipital gyrus (IOG). Remarkably, the amount of changed voxels at the third visit increased significantly (*N* = 616). The fourth and last visits showed significant increases in the right MPFC and right INS, and the changed voxel numbers gradually decreased during the last two visits (*N* = 135, *N* = 133). FWE correction was used for correcting the above results (*P* < 0.05). In Figure 5, clusters exhibiting significant FCS changes in each comparison are shown as bar charts. It should be noted that the sum of changed voxel numbers in each comparison first increased and then decreased over time, and the clusters showed FCS changes in the comparison between the patients at the <30 days visit, and the HCs contained more voxels than others. Detailed information is provided in Table 2. Moreover, in the validation test, our main findings were preserved (Supplementary Figure S1).

**TABLE 2:** Regions showing FCS differences between patients with five visits and HCs.

**Brain regions**	**BA**	**Peak MNI coordinates**			**Voxel number**	***T* value**
		**x**	**y**	**z**		
*<7 days – HCs*						
Right MPFC	11	33	57	−9	86	5.51
Right MFG	10	45	57	6	37	4.98
*14 days – HCs*						
Right MPFC	11	45	48	−15	82	5.25
Right MFG	10	54	30	15	55	4.38
*30 days – HCs*						
Right MPFC	11	24	63	−15	465	5.90
Right INS	47	33	25	4	53	4.10
Left MFG	10	−6	23	−12	21	4.63
Left CAL	17	−9	−66	12	7	−4.17
Right IOG	18	33	−84	−15	70	−4.90
*90 days – HCs*						
Right MFG	10	42	48	−15	108	4.19
Right INS	47	36	27	−6	37	4.35
*180 days – HCs*						
Right MFG	10	51	33	27	123	4.90
Right INS	47	33	57	0	10	5.11

*BA, Brodmann’s area; x, y, z, coordinate of primary peak locations in the MNI space; MFG, middle frontal gyrus; MPFC, medial prefrontal cortex; CAL, calcarine; LING, lingual gyrus; INS, insula; IOG, inferior occipital gyrus. *P* < 0.05, FWE corrected.*

### Lateralization Index Changes

**FIGURE 6:**
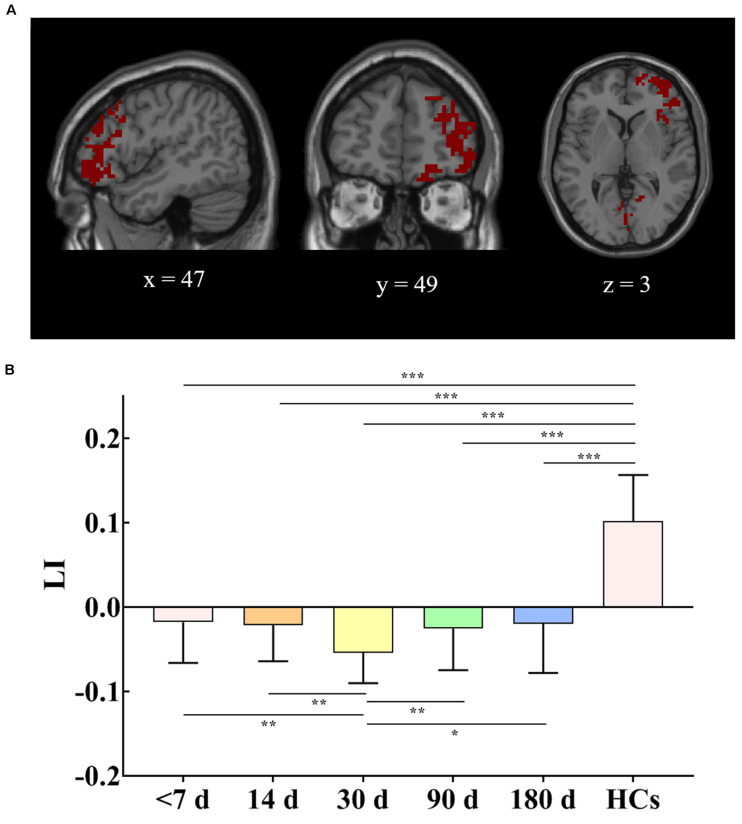
**(A)** Union of brain regions from five FCS comparisons between patients at five visits and HCs. **(B)** LI changes in patients at the last four visits compared with baseline and patients at five visits compared with HCs. The mask for LI calculation used the mirror image superimposed brain area, which is shown in **(A)**. Error bars show standard deviations. HCs, healthy controls; FCS, functional connectivity strength; LI, lateralization index. *P < 0.05, **P < 0.01, ***P < 0.001.

Intra- and between-group LI changes are shown in Figure 6. As seen from the figure, there was significant LI difference among the different visits in the patient group (*F* = 5.27, *P* = 0.01). The *post hoc* test revealed that the LI at the 30-day visit was significantly different from those at the other four visits in the patient group (*P* < 0.05), which showed a trend of first a decline and then an increase. Additionally, the LI of HCs (0.10 ± 0.05) showed left hemisphere dominance, while the LI values for the patients with five visits (<7 days, −0.01 ± 0.04; 14 days, −0.02 ± 0.04; 30 days, −0.05 ± 0.03; 90 days, −0.02 ± 0.04; 180 days, −0.01 ± 0.05) were negative and lost the left hemisphere dominance, which significantly differed from LI of the HCs (*P* < 0.001). Combined with the above results, it should be noted that the tendency of losing left hemisphere dominance in patients at the 30-day visit was significant (*P* < 0.05). The main findings about LI were preserved in the validation test (Supplementary Figure S2).

### Correlations Between Changes in Motor Function and Changes in Functional Measurements

No significant correlation was found between changes in functional measurements (ΔFCS and ΔLI) and changes in motor function (ΔFMA and Δgrip strength).

## Discussion

In this study, we constructed a voxel-based whole-brain functional network to estimate the FCS and LI and to analyze the longitudinal effects between functional measurements (FCS and LI) and motor function (FMA and grip strength) in the left BG stroke patients. We obtained the following results: (a) comparing the HCs, the BG stroke patients at five visits showed varying degrees of FCS. Major FCS decreases gathered in the left CAL and right IOG, and FCS increases more likely located in the right MPFC, bilateral MFG, and right INS. (b) Comparing the HCs, LI lost the left hemisphere dominance in patients, and LI changes at the 30-day visit were significant. (c) Transition of FCS and LI changes occurred in the patients with five visits, and the turning point occurred approximately 30 days after onset, which served as a predictive biomarker for the development of BG stroke. The findings suggested that the functional network in BG stroke patients showed strong plasticity and could be reorganized with different temporal dynamics.

### Dynamic Network Reorganization Showed a Transition at the 30-Day Visit

Most previous studies have confirmed the involvement of changes in the cortical functional domain during motor function recovery in stroke patients. However, there is no consensus on the correlation between cortical activation and motor functional rehabilitation, the activated site of the brain, activation intensity, the recovery process, and the efficiency of rehabilitation therapy. In this study, we documented the FCS and LI changes in the left BG patients using longitudinal functional MRI examination, which was able to delineate the development of the functional changes.

This longitudinal study indicated the trajectory of FCS and LI alterations, which showed the functional transition in BG stroke patients at the 30-day visit. The results were consistent with previous findings in both animal and human studies (Meer et al., 2010; Park et al., 2011; Zhang et al., 2016). Park et al. (2011) found that the lowest connectivity of the interhemispheric motor network appeared 1 month after the stroke, followed by a subsequent reincrease. Additionally, Zhang et al. (2016) reported increased FC between the bilateral primary motor cortex after 1 month of conventional rehabilitation and suggested that 1 month after onset in stroke patients was a critical period of rehabilitation. Moreover, Meer et al. (2010) demonstrated highly similar findings in rats. They used poststroke fMRI data from rats to explore the time course of connectivity changes and reported that the connectivity between the bilateral hemispheric sensorimotor areas decreased over the first 2 weeks (rats showed a relatively short reverse time than humans) and then reincreased alongside the recovery of sensorimotor function. Combined with the above findings, these data provide supports for the view that the prime time for treatment in BG stroke patients was within 30 days after onset and that the clinical doctor could proceed with interventional therapy using reliable rehabilitation methods during this critical time.

### Abnormal FCS Changes in Patients

Many functional imaging studies of stroke have reported the occurrence of dysfunction across brain areas, including the cortical regions of sensorimotor, temporal, parietal, and occipital areas (Ferrandez et al., 2003; Ward et al., 2003). By tracking detailed FCS alterations in BG stroke, this study showed both decreased and increased FCS in different cerebral regions across the five visits in the left BG stroke patients compared with the HCs. In the left BG stroke patients, we found that decreased FCS localized in the CAL and IOG. The results coincide with the previous task-based fMRI studies, which reported non-motor brain areas such as the occipital cortex are in association with motor tasks in patients with stroke (Ward et al., 2003). Moreover, Park et al. (2011) acquired rs-fMRI data of stroke patients with four visits and HCs, performed analysis of FC, and showed the stroke patients had decreased FC in the occipital cortex. In addition to motor dysfunction, BG stroke can also lead to impairments in executive function, visual memory, and other aspects. These disorders can cause complex changes in brain function involving multiple brain regions (Wang et al., 2017; Tambasco et al., 2018). Thus, our observation of decreased FCS in several occipital regions may contribute to these deficits.

Additionally, we also observed increased FCS localized in the right MPFC and right INS in the BG stroke patients, which agreed well with the findings of Fan et al. (2015) and Zheng et al. (2016). The MPFC has not been regarded as a primary or secondary sensorimotor region but as a region that is associated with high-level executive functions and decision-related processes (Zheng et al., 2016). Moreover, it has been indicated that the INS was a participant in motor control and cognitive functioning (Wang X. et al., 2014). Thus, increased FCS in the MPFC and INS may imply that motor and cognitive manipulation had become stronger because of the shortage of motor output affected by the infarction lesion (Fan et al., 2015). It should be noted that those increased regions were primarily localized in the contralesional hemisphere, which was taken as the compensatory mechanism to compensate for the motor impairment caused by the damaged brain region (Zheng et al., 2016; Desowska and Turner, 2019).

### LI Changes in Patients

The breakdown of harmonious interaction could be quantitatively characterized in terms of the LI, which was calculated based on the union of regions with altered FCS in this study. Coinciding with the perspective of Thiel et al. (2006) and Bartolomeo and Thiebaut de Schotten (2016), the healthy hemisphere could be integrated into brain function and compensate for the lost brain function of the damaged hemisphere. Our results of the LI showed that corresponding regions of FCS changes in the stroke patients were highly lateralized to the contralesional hemisphere, suggesting compensation from the contralesional hemisphere. Therefore, the combined results of the current study suggest that the breakdown of harmonious interactions between the bilateral cerebral hemispheres may have led related brain regions to undergo neurophysiological inhibition and compensation. These findings also implied that FCS in the non-affected area was indicative of the disease dynamic of cerebral stroke.

### Limitations

Limitations did exist in the current study and should be acknowledged. First, although the current study enrolled only patients with a single left BG stroke lesion, further research might take the relative heterogeneity in lesion region or volumes into consideration. Second, the participants in this study did not adequately assess the association between functional patterns and clinical neurophysiological scales. Although the current study has described longitudinal changes in motor function and calculated the longitudinal effects between brain function and behavioral assessments, there was no significant correlation. Thus, the correlations between functional pattern and neurophysiological information still require further exploration. We suggested that in the subsequent studies on BG stroke patients, besides the motor function scale, researchers also need to measure the cognitive function scale to help us further understand the brain function mechanism of stroke. Third, one-way ANOVA analysis of FCS in patients had no significant results, which may due to the insufficient sample size. On this condition, we performed between-group comparisons between patients during five visits and HCs and found that the variation size trend of significant FCS cluster obtained in five between-group comparisons is consistent with the longitudinal comparison trend of LI. So, we suggested that although FCS lacks the results of ANOVA analysis, the above results of between-group analysis and LI may indirectly reflect the longitudinal FCS changes in patients. Additionally, future studies should collect more samples to further verify our results. Fourth, the study examined functional changes using only rs-fMRI, while recent studies have indicated that reorganization of brain function was also related to damage to brain structure (Liu J. et al., 2015; Thiel and Vahdat, 2015; Desowska and Turner, 2019). Thus, to better understand the neural mechanisms of reorganization, further studies are required to combine FC with structural studies.

## Conclusion

The current study examined voxel-based FCS as well as LI in left BG stroke patients across five visits. We found that decreased FCS localized in the CAL and IOG and increased FCS localized in the MPFC, MFG, and INS in the BG stroke patients. Furthermore, our results revealed that left BG patients exhibited a transitional variance in LI and FCS changes at the 30-day visit. The results of the current study provide not only new evidence of the neural effects on the voxel-based whole-brain network but also an indication that the critical convalescence stage in BG stroke patients was approximately 30 days after onset.

## Data Availability Statement

All datasets generated for this study are included in the article/Supplementary Material.

## Ethics Statement

The studies involving human participants were reviewed and approved by the Ethics Committee of Xuanwu Hospital of Capital Medical University. The patients/participants provided their written informed consent to participate in this study.

## Author Contributions

JL conceived and designed the study and reviewed and edited the manuscript. MZ, D-DR, and Q-FM recruited the subjects. CZ and YS performed the experiments. Q-GL and Y-YY analyzed the data. Q-GL and CZ wrote the manuscript. All authors contributed to the article and approved the submitted version.

## Conflict of Interest

The authors declare that the research was conducted in the absence of any commercial or financial relationships that could be construed as a potential conflict of interest.

## Footnotes

1 www.nitrc.org/projects/mricron
