# Supplementary material for: Dynamic Neural Network Changes Revealed by Voxel-Based Functional Connectivity Strength in Left Basal Ganglia Ischemic Stroke
Source: Front Neurosci. 2020 Sep 18;14:526645. doi: 10.3389/fnins.2020.526645 (PMC7533550; doi:10.3389/fnins.2020.526645)
Supplement: Supplementary file 1 [file Table_1.DOCX]

Supplementary Material

# Supplementary Figures


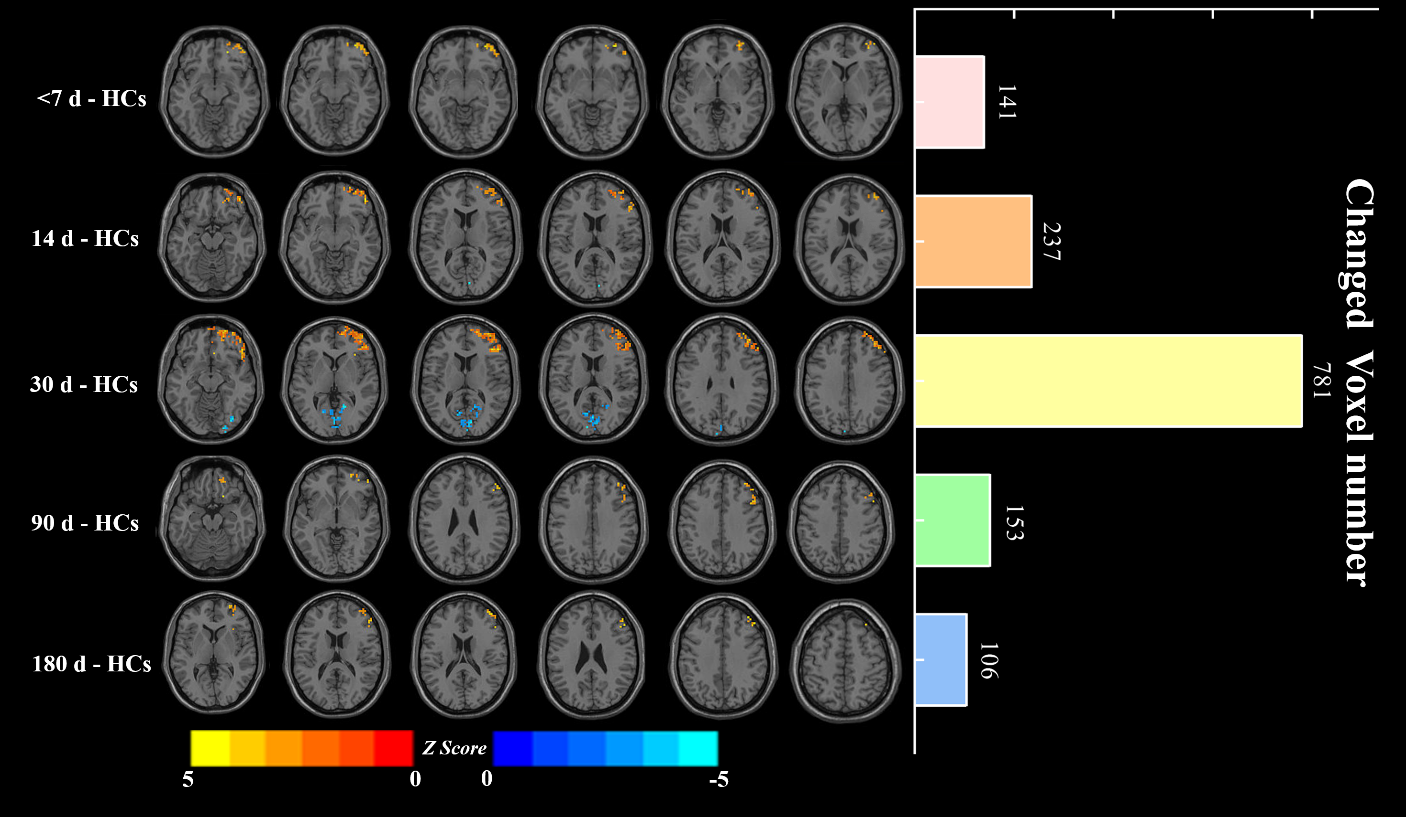


**Figure S1.** Significant FCS differences (r=0.4) and changed voxel numbers between patients at five visits of <7 d, 14 d, 30 d, 90 d, 180 d and HCs. Axial maps of each row represent results of each contrast. Values are expressed as z-scores, where hot colors represent increased FCS and cold colors represent decreased FCS in the left BG stroke patients. Each item in the right bar graph corresponds to each row in the left axis map. Decreased FCS localized in the CAL and IOG, and increased FCS localized in the right MPFC, MFG and right INS in the BG stroke patients. And left BG patients exhibited a transitional variance in FCS changes at the 30-d visit. FCS, functional connectivity strength; HCs, healthy controls.


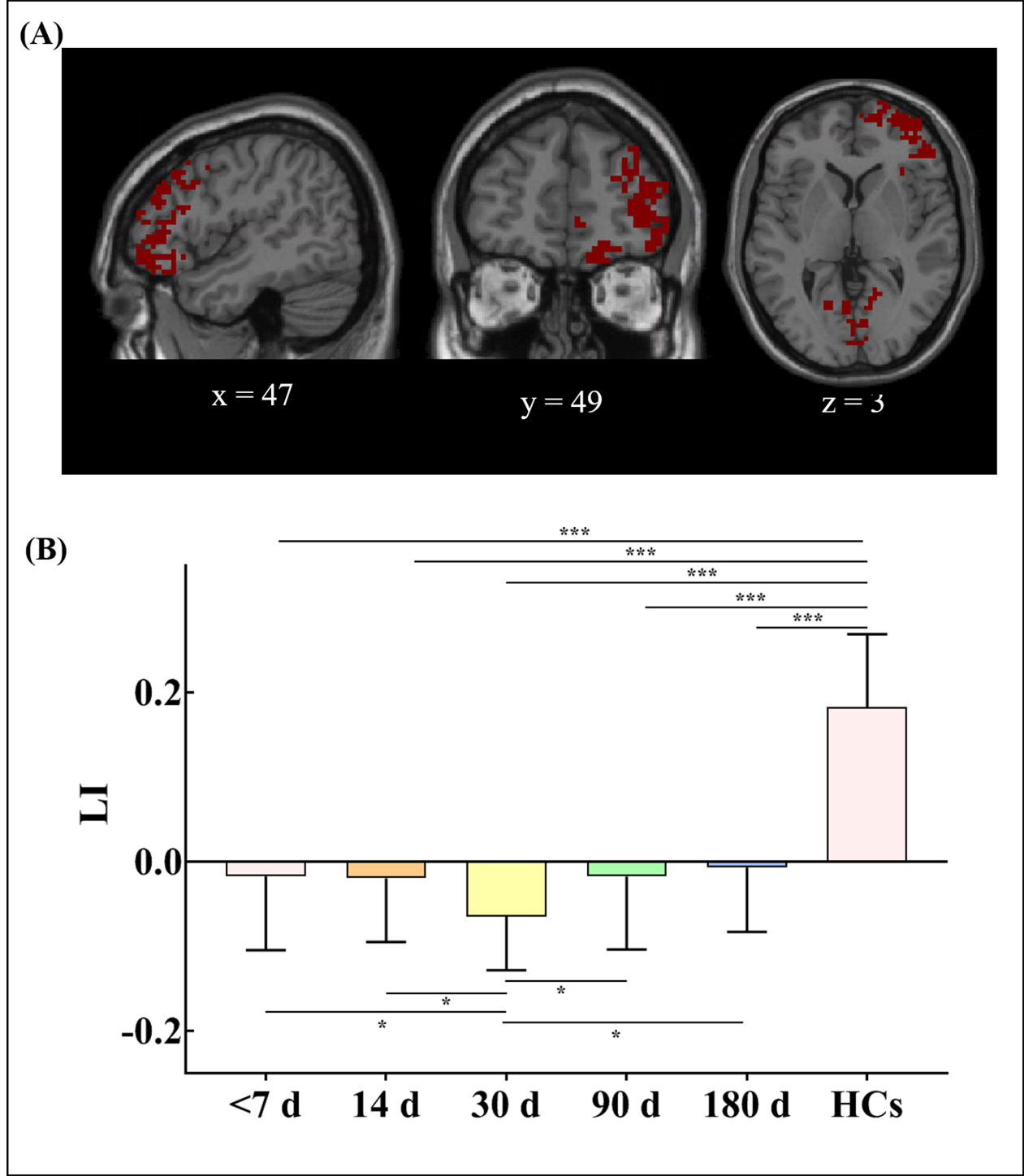


**Figure S2.** (A) Union of brain regions from five FCS comparisons (r=0.4) between patients at five visits and HCs. (B) LI changes in patients at the last four visits compared with baseline and patients at five visits compared with HCs. The mask for LI calculation used the mirror image superimposed brain area, which is shown in A. Error bars show standard deviations. LI of HCs showed left hemisphere dominance, while the LI of the patients with five visits approached zero and lost the left hemisphere dominance, and the tendency of losing left hemisphere dominance in patients at the 30-d visit was significant. HCs, healthy controls; FCS, functional connectivity strength; LI, lateralization index. *, P<0.05; ***, *P*<0.001.
